# Characteristic visuomotor influences on eye-movement patterns to faces and other high level stimuli

**DOI:** 10.3389/fpsyg.2015.01027

**Published:** 2015-07-29

**Authors:** Joseph M. Arizpe, Vincent Walsh, Chris I. Baker

**Affiliations:** ^1^Applied Cognitive Neuroscience Group, Institute of Cognitive Neuroscience, University College LondonLondon, UK; ^2^Section on Learning and Plasticity, National Institute of Mental Health, National Institutes of HealthBethesda, MD, USA; ^3^Department of Neurology, University of Tennessee Health Science CenterMemphis, TN, USA; ^4^Pediatrics Department, Le Bonheur Children’s HospitalMemphis, TN, USA

**Keywords:** eye movements, distance effect, face recognition, object recognition, visual perception, visual fields, gaze control, visuomotor control

## Abstract

Eye-movement patterns are often utilized in studies of visual perception as indices of the specific information extracted to efficiently process a given stimulus during a given task. Our prior work, however, revealed that not only the stimulus and task influence eye-movements, but that visuomotor (start position) factors also robustly and characteristically influence eye-movement patterns to faces (Arizpe et al., 2012). Here we manipulated lateral starting side and distance from the midline of face and line-symmetrical control (butterfly) stimuli in order to further investigate the nature and generality of such visuomotor influences. First we found that increasing starting distance from midline (4°, 8°, 12°, and 16° visual angle) strongly and proportionately increased the distance of the first ordinal fixation from midline. We did not find influences of starting distance on subsequent fixations, however, suggesting that eye-movement plans are not strongly affected by starting distance following an initial orienting fixation. Further, we replicated our prior effect of starting side (left, right) to induce a spatially contralateral tendency of fixations after the first ordinal fixation. However, we also established that these visuomotor influences did not depend upon the predictability of the location of the upcoming stimulus, and were present not only for face stimuli but also for our control stimulus category (butterflies). We found a correspondence in overall left-lateralized fixation tendency between faces and butterflies. Finally, for faces, we found a relationship between left starting side (right sided fixation pattern tendency) and increased recognition performance, which likely reflects a cortical right hemisphere (left visual hemifield) advantage for face perception. These results further indicate the importance of considering and controlling for visuomotor influences in the design, analysis, and interpretation of eye-movement studies.

## Introduction

The locations of fixations are commonly regarded as spatial indices of the information used to process given stimuli or perform given tasks, as it is assumed that the specific stimuli and the task are the primary determinants of the fixation pattern rather than, for example, visuomotor factors. However, in a previous study (Arizpe et al., 2012), we found that throughout at least the first five fixations to face stimuli, a non-stimulus, non-task factor, namely the pre-stimulus start position, had a robust impact on the location of fixations. For peripheral start positions (above, below, left of, and right of the upcoming face), the first fixation was likely the result of a simple initial localizing saccade as it was qualitatively different from subsequent fixations having a shorter duration and having a different spatial distribution compared to subsequent fixations, landing near the center of face with a slight tendency toward the start position. Notably though, on subsequent fixations, this spatial tendency flipped to one strongly tending on the side of the face opposite the start position. Those results indicate that the absolute locations of fixations during face processing can be strongly influenced by factors beyond stimuli and task, reflecting influences of visuomotor effects. Our results, critically, suggested that previously reported fixation patterns based on a single start position or the average across multiple start positions may not accurately reflect the information used in face processing.

In the present study, we first sought to further investigate potential sources of such non-stimulus, non-task visuomotor influences on eye-movements by manipulating lateral distance of the start position from the upcoming stimulus (4°, 8°, 12°, and 16° of visual angle from midline), with the principal aim of determining whether lateral starting distance from a face impacts subsequent eye movement patterns. To our knowledge, no prior published studies have investigated the influence of starting distance on eye-movements to high level stimuli; however, there are previous reports of a systematic saccadic range error for word and simple point stimuli (Kapoula, 1985; Kapoula and Robinson, 1986; McConkie et al., 1988; Radach and McConkie, 1998, but see Vitu, 1991). In light of those reports, and taking the midline of our stimuli as the reference, we specifically hypothesized that for the first ordinal fixation we would find an overshoot of the midline for near stimuli and an undershoot for far stimuli. Additionally, given the results of our prior study (Arizpe et al., 2012), fixations subsequent to the first ordinal fixation were expected to show a tendency opposite to the start position, though we were also interested in any evidence that starting distance could modulate the strength of this tendency. Given that what is of interest in most eye-tracking studies is how stimulus and/or task influence eye-movement patterns, a clear characterization of the influences of non-stimulus, non-task factors, such as starting distance, can be informative for the design, analysis, and interpretation of eye-tracking studies so that artifactual fixation pattern effects are not confounded with effects of interest. Further, faces in real-life typically appear in peripheral vision and require an initial saccade to bring them close to the fovea, so the impact of starting distance from the face is important for understanding eye movements to faces.

Second, to extend and confirm the findings from our prior study, we also manipulated the relative location of the start position to the stimulus (left and right side). In particular, given that the approximate location of the upcoming face stimulus relative to the start position was predictable in our prior study, we instead utilized a paradigm rendering the location of the upcoming stimuli unpredictable so as to test our hypothesis that the visuomotor influences induced by the start position also apply when predictability is greatly reduced.

Third, to determine whether any visuomotor effects are specific to face stimuli, the present study also utilized butterfly stimuli as line symmetrical control stimuli. We hypothesized that visuomotor influences are not specific to stimulus category.

Last, informed by a trend observed in our prior study we also tested a hypothesis that differences in fixation patterns associated with starting side, and possibly also distance, relate to recognition performance. Specifically, we hypothesized that left start position (which induces a right sided fixation tendency) is associated with higher recognition performance.

We observed strong effects of Distance on the first ordinal fixation, with an increasing undershoot of the midline of the stimulus with increasing distance. Notably, there was no strong impact on later fixations. These effects of Distance, as well as the previously reported effects of left and right starting side, were not specific to faces. We also established that the subsequent contralateral tendency in fixation patterns does not depend on predictability of the location of the upcoming stimulus or on stimulus category (faces and butterflies). We observed a correspondence between face and butterfly stimuli in overall left laterality in fixation tendency, indicating that left lateral tendency in fixation patterns is not specific to face perception. Lastly, we found a relationship between left starting Side (right-sided fixation pattern tendency) and increased recognition performance for faces. We discuss what the methodological implications for eye-tracking studies and the mechanistic implications for visual perception are given the visuomotor influences we report.

## Materials and Methods

### Ethics Statement

All participants gave written informed consent and were compensated for their participation. The study was approved by the Institutional Review Board of the National Institutes of Health, Bethesda, MD, USA.

### Participants

We recruited 17 right-handed participants, living in the Washington D.C. area. Three were excluded because of poor eye-tracking calibration or because of unusually rapid pace through the experiment resulting in very few fixations on each face. Thus, 14 participants (six male) are included in analyses.

### Eye-Tracking

We used an EyeLink II head-mounted eye-tracker (SR Research, Mississauga, ON, Canada), and sampled pupil centroid at 250 Hz. Participants’ eyes were 57 cm from the stimulus display screen. The default nine point calibration and validation sequences were repeated throughout the experiment. Both eyes were calibrated and validated, but only the eye with the lowest maximum error was recorded for the trials following a particular calibration. Calibration was repeated when maximum error at validation was more than 1.33° of visual angle. Average validation error was always substantially lower than 1° of visual angle. The mean of the average validation errors was 0.35° of visual angle with a standard deviation of 0.086° of visual angle. The mean of the maximum validation errors was 0.86° of visual angle with a standard deviation of 0.22° of visual angle. To minimize head motion artifacts, all participants had their heads fixed with a chin rest and, additionally, the “Head Camera” feature of the EyeLink II was engaged so as to provide some compensation for head motion that could still occur. Further, before each trial, a drift correction was performed. Default criteria for fixations, blinks, and saccades as implemented in the Eyelink system were used.

### Stimuli

**FIGURE 1:**
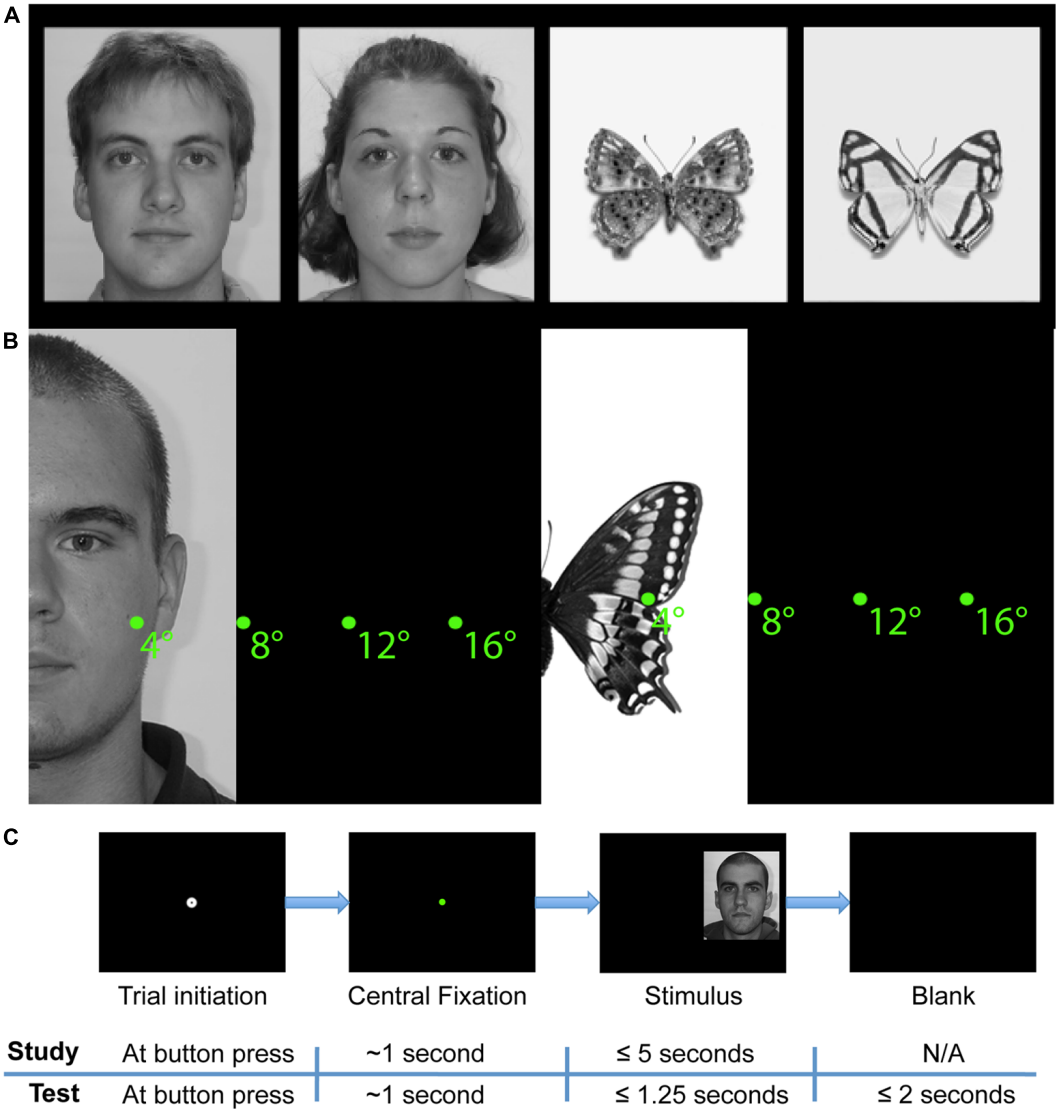
**Study design. (A)** Example face and butterfly stimuli. **(B)** Schematic depiction of starting distances from midline of stimuli. **(C)** Schematic of trial sequences in study and test phases. A stimulus was presented only if the participant successfully maintained fixation for a total of 1 s. After stimulus onset in the study phase, participants were free to study the face for up to 5 s and pressed a button to begin the next trial. In the test phase, faces were presented for 1.25 s only and participants responded with button presses to indicate whether the stimulus was ‘old’ or ‘new.’

We collected 96 Caucasian-American (48 male) grayscale neutral expression frontal-view face images (see **Figure 1A** for examples). All face images were taken from the neutral expression 18–29 age group of the Productive Aging Lab Face Database established by the University of Texas at Dallas^1^ (Minear and Park, 2004). Each face was scaled to have a forehead width subtending 10° of visual angle at presentation and was rotated to correct for any tilt of the head. Images were cropped to remove most of the background, but not the hair or other external features, and all face images were equated for overall luminance. At presentation, images were centered on a black background. To eliminate any possible stimulus bias as the source of any laterality effects, half of the faces were randomly left–right flipped across the vertical midline of the image for each participant. The website of the Productive Aging Lab Face Database states: “This [database] contains a range of face of all ages which are suitable for use as stimuli in face processing studies. Releases have been signed by the participants we photographed and the faces may be included in publications or in media events.”

We also collected 96 grayscale “butterfly” images each of a pinned specimen of a unique species of Lepidoptera on a white background. All butterfly images were taken from Butterflies of America^2^ , a website devoted to the study and enjoyment of American butterflies (Warren et al., 2013). The butterflies were aligned at the convergence of the upper and lower wings, and scaled so that the maximum width of the upper wing close to the point of alignment was the same. This width matched the width of the faces. As with the faces, the butterfly images were cropped to remove most of the background and were equated for overall luminance. Likewise, at presentation, butterfly images were also centered on a black background and half were randomly left–right flipped across the vertical midline of the image for each participant to eliminate any possible stimulus bias as the source of any laterality effects.

### Areas of Interest (AOIs)

To aid alignment of the face images and positioning relative to the fixation starting position, rectangular areas of interest (AOIs) were manually drawn uniquely for each face around the right and left eyes, bridge of nose (i.e., middle of eye region), right and left half of nose, and right and left half of mouth (Supplementary Figure S1, for example) using EyeLink Data Viewer software. These AOIs were never visible to participants during the experiment.

### Design

The experiment comprised of two parts, one with face stimuli and the other with Lepidoptera (“butterfly”) stimuli. Both parts were completed within the same experimental session. Each part had two phases: study and test. During the study phases, participants observed 48 faces (24 female) or 48 butterflies (each of a unique Lepidoptera species) in a self-paced manner (up to 5 s, self-terminating trials with a button press). At test, participants observed 96 faces or butterflies (the 48 study phase faces or butterflies plus 48 new faces or butterflies) for a limited duration (1250 ms limit) and indicated with a button press whether or not they recognized each stimulus (old/new task) as one observed during the study phase. Participants were given up to 3 s to respond following stimulus onset and were instructed to respond as soon as they thought they knew the answer (**Figure 1C**) and to guess if they were not sure. The experiment was programmed in Python and interfaced with the eye-tracker.

Across trials, we systematically varied two factors (i) the side of the visual field that the face or butterfly appeared relative to the central fixation dot at the beginning of each trial, which thus varied the start position (“Side”) relative to the face or butterfly, and (ii) the distance along the horizontal-axis from midline of the face or butterfly stimulus (“distance”) relative to the same starting fixation dot. We varied left and right side because fixation patterns are affected by visuo-motor factors (e.g., start position) in addition to stimulus factors (e.g., face), (Arizpe et al., 2012). Note that the manner in which side (start position) was varied in the present study differs from our prior study (Arizpe et al., 2012). Specifically, in the current study, we accomplished this by varying the side that the face appeared relative to a central starting fixation dot, whereas in the prior study we accomplished this by varying the location of the starting fixation dot relative to the centrally presented face stimuli. This difference in paradigm aimed to induce greater uncertainty about the location of the upcoming stimulus, and thus to allow us additionally to test whether the effects of start positions previously observed also occur under greater uncertainty about the stimulus location.

Side was defined in terms of the start position, so ‘left’ refers to starting on the left of the face, not to a face presented in the left visual field. In the case of faces, position along the *y*-axis of the screen was calculated uniquely for each face stimulus such that the central starting fixation dot would always have the same *y*-coordinate component as the unique point equidistant from all of the nearest internal facial features. Specifically, that unique coordinate was calculated numerically for each face such that it was equidistant from the centers of the nearest eye, nearest half-nose, and nearest half-mouth AOI. In the case of butterflies, the *y*-coordinate of each butterfly stimulus was selected such that the starting fixation dot was always at the same *y*-coordinate as the convergence of the upper and lower wings of the butterfly. Distance varied such that the midline of the face or butterfly was 4°, 8°, 12°, or 16° of visual angle from the starting fixation dot along the *x*-axis (see **Figure 1B** for examples, see Supplementary Figures S2–S7 for full screen examples and schematics).

Before stimulus onset, participants fixated the start position at the center of the screen, indicated by a standard EyeLink II calibration target (0.17° diameter black circle overlaid on a 0.75° diameter white circle) on the black screen. Participants initiated the trial by pressing a button while looking at the fixation target. In this action, a drift correction was performed. A colored dot (0.5° diameter) remained after drift correction, and the stimulus appeared only after the participant had fixated the dot for an accumulated total of 750 ms. This process ensured that drift correction and fixation were stable prior to stimulus onset. If more than 750 ms of fixation away from the start position accumulated before the trial could be initiated, drift correction was repeated. A fixation was considered off the start position if it landed more than 0.5° from the center of the dot. Dot color changed successively from red to yellow to green in order to signal to the participant that a maintained fixation was successfully detected at the start position.

In both the study and test phases, there were equal proportions of trials for each combination of levels of the factors of side, distance, and in the case of the face stimuli, face gender. The particular subset of faces and butterflies used in the study phases was randomized across participants. Of the faces and butterflies presented in both study and test phase, all were presented on the same side of the visual field and the same Distance condition at study and test. The order in which the face and butterfly parts of the study were run was counterbalanced across subjects.

### Analyses

#### Software

Fixation and AOI data were obtained through EyeLink Data Viewer software by SR Research. Subsequent analyses on these data and behavioral data from the test phase were performed with custom Matlab (The MathWorks, Inc., Natick, MA, USA) code. Statistical tests were performed in SPSS (IBM, Somers, NY, USA).

#### Behavior

We assessed participants’ discrimination performance, response bias, and reaction time on the old/new recognition task in the test phase. *d*′ (*d*′ = *z*(hit rate) – *z*(false alarm rate)) and criterion *c* (*c* = –[*z*(hit rate) + *z*(false alarm rate)]/2) were computed for discrimination performance for each participant, broken down by Stimulus Category, Side, and Distance. Reaction times were analyzed for correct trials only. Reaction time analyses were also broken down by start position and stimulus conditions with analysis being performed on the medians calculated for each participant. Medians, rather than means, were calculated for each participant (as is common practice for reaction time analyses) because reaction time distributions tend to be skewed to high reaction times. The mean reaction times displayed in our figure are the means of the participant medians.

#### Spatial Density Analyses

We mapped the spatial density of fixations during the study phase as a function of our experimental manipulations. Each fixation was plotted with equal density and spatial extent, as fixations were not weighted by the fixation duration. Fixations beyond the fifth fixation were excluded from the analysis to ensure an equal amount of data across trials. To ensure that summation of fixation maps across different face trials produced spatially meaningful density maps, fixation maps for individual faces were first aligned to a common reference frame using simple translations only. The internal facial features defined this reference frame. Specifically, the alignment minimized the sum of the squared differences between the center of the AOIs for each face and the average centers of the AOIs across all 96 faces. For the same purpose, fixation maps across different butterfly trials were first aligned such that the line of convergence between the top and bottom wing coincided across stimuli. All stimulus images had already been scaled to be comparable size, so rescaling was not necessary in order to align fixation maps.

Within this common reference frame, fixations were then plotted as Gaussian densities with the peak density over the fixation coordinate and a SD of 0.3° of visual angle in both the *x*- and *y*-dimensions. These density plots were then averaged across trials and across participants. The negligible proportion of fixations (<1.1% during study phase) that fell outside of the bounds of the stimulus image analysis region (i.e., onto the black background outside the square frame of the face or butterfly stimulus) were shifted to the nearest edge within the analysis region so that total fixation density was comparable across analyses. The resulting maps show the spatial fixation densities, using a color scale from zero to the maximum density value observed, with values approaching zero being deep blue. All maps within a single figure contain the same total number of fixations and so are scaled the same to allow for direct comparison.

#### Profile Density Analyses

We calculated profile densities (i.e., densities summed along a single dimension of a heatmap) for the different conditions during the study phase. The *x*-profile plots were produced by summing along the vertical dimension (*y*-axis) of a spatial density heatmap, and *y*-profile plots were produced by summing along the horizontal dimension (*x*-axis) of a spatial density heatmap. The *x*-profile plots visualize the overall left–right laterality of fixations. The *y*-profile plots visualize fixation density over specific vertical (e.g., facial) features without respect to laterality or fine differences in horizontal position. Since the main focus of this study was to determine the laterality of fixations with respect to the midlines of our stimuli, we largely focused on *x*-profile plots.

#### Similarity Matrix Analyses

In order to quantify and visualize the degree of similarity between fixation patterns among the different side and distance conditions, we computed similarity matrices for the spatial density data. This data visualization method allows for concise visualization of the relative similarities in overall data patterns between given conditions, and enables further quantitative analysis on the relative degrees of similarity. A similarity matrix simply is an organized matrix, in which each cell represents a comparison between given conditions, or a given combination of conditions, and contains a value of a specific similarity measure (e.g., correlation value, Euclidean distance, etc.) corresponding to that specific comparison, which is specified by its index in the matrix. This class of methods, along with the complementary class of discrimination analyses (see “Discrimination Analyses” subsection below), has become common in fMRI studies (e.g., Haxby et al., 2001; Kriegeskorte et al., 2008) and has also been used in prior eye-tracking studies (Benson et al., 2012; Greene et al., 2012; Tseng et al., 2013; Borji and Itti, 2014), including two on face perception (Mehoudar et al., 2014; Kanan et al., 2015).

In our study, the possible combinations of conditions for which fixation patterns were compared between were the levels of the factors of ordinal fixation number (1–5), side (left, right), and distance (4°, 8°, 12°, 16° visual angle) separately for each stimulus type (faces, butterflies). In our analyses, only the study phase data were utilized since the test phase had a limited duration (1250 ms limit) that the stimulus was on the screen, thus limiting the number of fixations possible to analyze and also potentially inducing a pattern of eye-movement dynamics different from those of the study phase due to the time constraints of the recognition task. For our analysis, we conducted “split-half” analyses in which eye-movement data was first split into two halves, namely, the first and last 24 trials of the study phase, as each half had equal numbers of all possible combinations of conditions (side, distance, and face gender). Spearman’s correlations between corresponding (i.e., located at identical spatial coordinates) pixels’ density values across the split halves of the data were calculated for each given comparison for each subject, and, only when being visualized (i.e., in our figures), the values were then averaged across subjects. For similarity analyses in which the levels of a factor (e.g., left and right side) were pooled together, correlations were first computed between each half of the data containing identical levels of the pooled factor, and then values were averaged across levels.

The use of Spearman’s correlations, rather than Pearson’s correlation, for producing such pattern similarity measures is considered best practice (e.g., Rousselet and Pernet, 2012). Further, in the context of our study, in which fixation density patterns are correlated with one another, we knew *a priori* that our data would not technically satisfy all of the assumptions of the Pearson’s correlation. Principally this is because the distribution of fixation density values across the pixels of each heatmap can almost be guaranteed not to have a normal distribution, but rather a skewed distribution, owing to the fair number of pixels with density values at or near zero in the spaces where fixations did not tend to land (i.e., away from the internal features of the face or butterfly).

#### Discrimination Analyses

In order to quantify and test the significance of the average distinctiveness (“discriminability”) of the patterns of given conditions compared to those of other conditions, we conducted several discrimination analyses using the correlation values from the similarity matrix analyses. We particularly focused on discriminability among the levels of the distance factor. In the similarity matrices, the diagonals always corresponded to the correlation between the two halves of the data for the same condition, and the off diagonal cells to those of non-identical conditions; therefore, for each subject discriminability of each given condition was quantified as the mean difference between the diagonal and off diagonals for each row of the given similarity matrix, where along rows are all the given conditions for the first half (i.e., first 24 trials) of the data, and along columns are those of the second half (i.e., second 24 trials) of the data. Thus a discriminability value existed for each given condition and for each subject, in which greater positive values indicate greater relative discriminability. When testing for statistical significance of average discriminability for a given condition from the others, a one-tailed, one-sampled *t*-test of difference from zero was conducted on the discrimination index distribution across subjects for the given condition (row). A one-tailed test was chosen because only positive discrimination values are interpretable in that context. Note also that this is equivalent to a within-subject test, since discrimination indices were computed within subject.

## Results

### Temporal Dynamics of Eye-Movements

**FIGURE 2:**
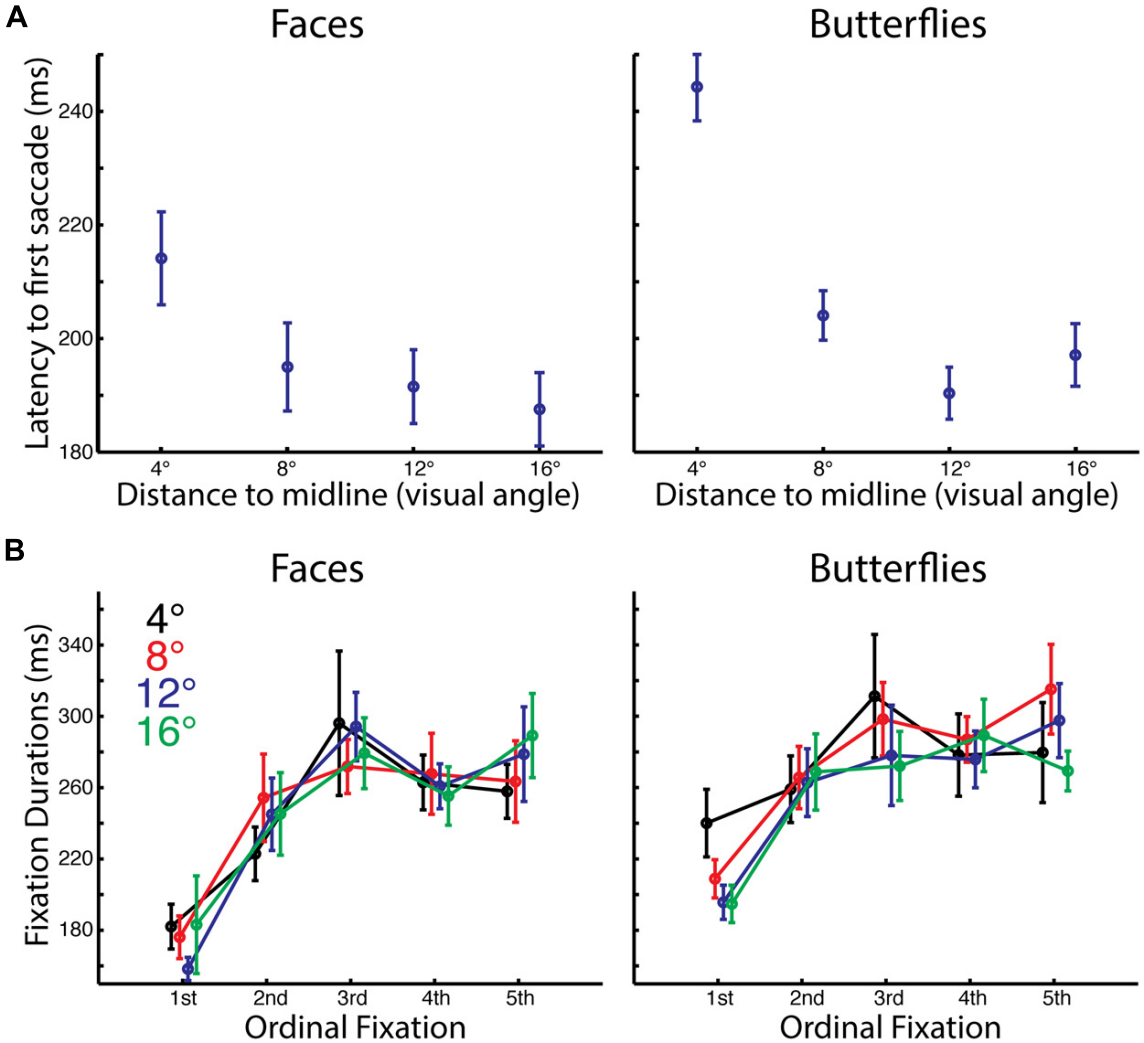
**Impact of starting distance on timing of initial saccades and fixations. (A)** Average latency to first saccade by starting distance and stimulus category. There was a longer delay between stimulus onset and the first saccade for the 4° distance compared to farther starting distances. Also there was a longer delay for butterflies than faces for the 4° distance. **(B)** Average duration of each of the first five fixations by starting distance and stimulus category. The first fixations were significantly shorter than subsequent fixations, and overall fixations were longer for butterflies than for faces.

We first investigated the temporal dynamics of eye-movements (**Figure 2**) to test for influences of Distance and Stimulus, as well as to test for replication of our prior findings (Arizpe et al., 2012) that revealed an influence of Ordinal Fixation, but not of left and right Side (start position), on the durations of fixations.

#### Latency to First Saccade

For both faces and butterflies, the latency to the first saccade was longer for the shortest distance than for the other distances. A three-way ANOVA with Stimulus Category (faces, butterflies), Distance (4°, 8°, 12°, 16° visual angle from stimulus midline), and Side (left, right) as within-subject factors revealed a main effect of Distance [*F*(3,39) = 54.65, *p* < 0.0005, $\eta_{p}^{2}$ = 0.81] and an interaction of Distance with Stimulus [*F*(3,39) = 7.31, *p* < 0.001, $\eta_{p}^{2}$ = 0.36]. A main effect of Stimulus approached significance [*F*(1,13) = 4.42, *p* < 0.057, $\eta_{p}^{2}$ = 0.25]. While *t*-tests on latencies with Side conditions pooled confirmed that for both faces and butterflies there was a longer latency for the shortest Distance than the longer distances [all paired *t*(13) > 3.33, *p* < 0.0055, two-tailed, bias corrected *g*_Hedges_ > 0.59], for butterflies only the latency at 12° was significantly shorter than that at 8 [paired *t*(13) = 3.073, *p* < 0.01, two-tailed, bias corrected *g*_Hedges_ = 0.76]. Additionally, there was a longer latency for butterflies than faces for the shortest Distance only [paired *t*(13) = 3.38, *p* < 0.0048, two-tailed, bias corrected *g*_Hedges_ = 1.05].

The increased latency for the first saccade at the shortest distance, when the starting position was already on part of the stimulus, likely reflects that even before initiating a saccade, our participants were already processing the stimulus information more deeply.

#### Fixation Durations

For both faces and butterflies, fixation duration tended to increase with ordinal fixation number. Also, overall fixation durations were longer for butterflies than for faces A four-way ANOVA on fixation durations with Stimulus Category (faces, butterflies), Distance (4°, 8°, 12°, 16° visual angle from stimulus midline), Ordinal Fixation (1st, 2nd, 3rd, 4th, 5th) and Side (left, right) as within-subject factors revealed main effects of Stimulus Category [*F*(1,13) = 7.99, *p* < 0.015, $\eta_{p}^{2}$ = 0.38] and Ordinal Fixation [*F*(4,52) = 17.93, *p* < 0.0005, Greenhouse–Geisser corrected, $\eta_{p}^{2}$ = 0.58], but no other main effects or interactions (all, *p* > 0.12, Greenhouse–Geisser corrected, $\eta_{p}^{2}$ < 0.15). Thus, Stimulus Category and Ordinal Fixation seem to have independently influenced fixation durations. The main effect of Stimulus Category was driven by overall longer duration fixations for butterflies than faces. *T*-tests on fixation durations pooling Stimulus Category, Distance, and Side revealed that the main effect of Ordinal Fixation was driven by a shorter first fixation than all the later fixations [all paired *t*(13) > 4.82, *p* < 0.0005, one-tailed, bias corrected *g*_Hedges_ > 1.29], as in our prior study (Arizpe et al., 2012). Durations of later Ordinal Fixations were not significantly different from each other [all paired *t*(13) < 1.65, *p* > 0.12, two-tailed, bias corrected *g*_Hedges_ < 0.36], except the second fixation was shorter in duration than the third fixation [paired *t*(13) = 3.30, *p* < 0.007, two-tailed, bias corrected *g*_Hedges_ = 0.51] and approached significance for being shorter than the fifth fixation [paired *t*(13) = 2.02, *p* < 0.066, two-tailed, bias corrected *g*_Hedges_ = 0.50].

The longer fixation durations for butterflies than for faces suggests perhaps that a different kind or depth of information processing was necessary for butterflies than for faces, consistent with the reduced accuracy for butterflies.

### Fixation Patterns

We used a number of different methods to examine fixation patterns and the effects of Category, Distance, and Ordinal Fixation number. We will first describe the spatial density profiles and differences in the overall patterns of fixations, computing similarity matrices across conditions. Then, since the main question of this study was the effect of Distance, which was manipulated in the *x*-direction only, we will focus on quantitative analyses of the distribution of fixations along this dimension.

#### Spatial Density

**FIGURE 3:**
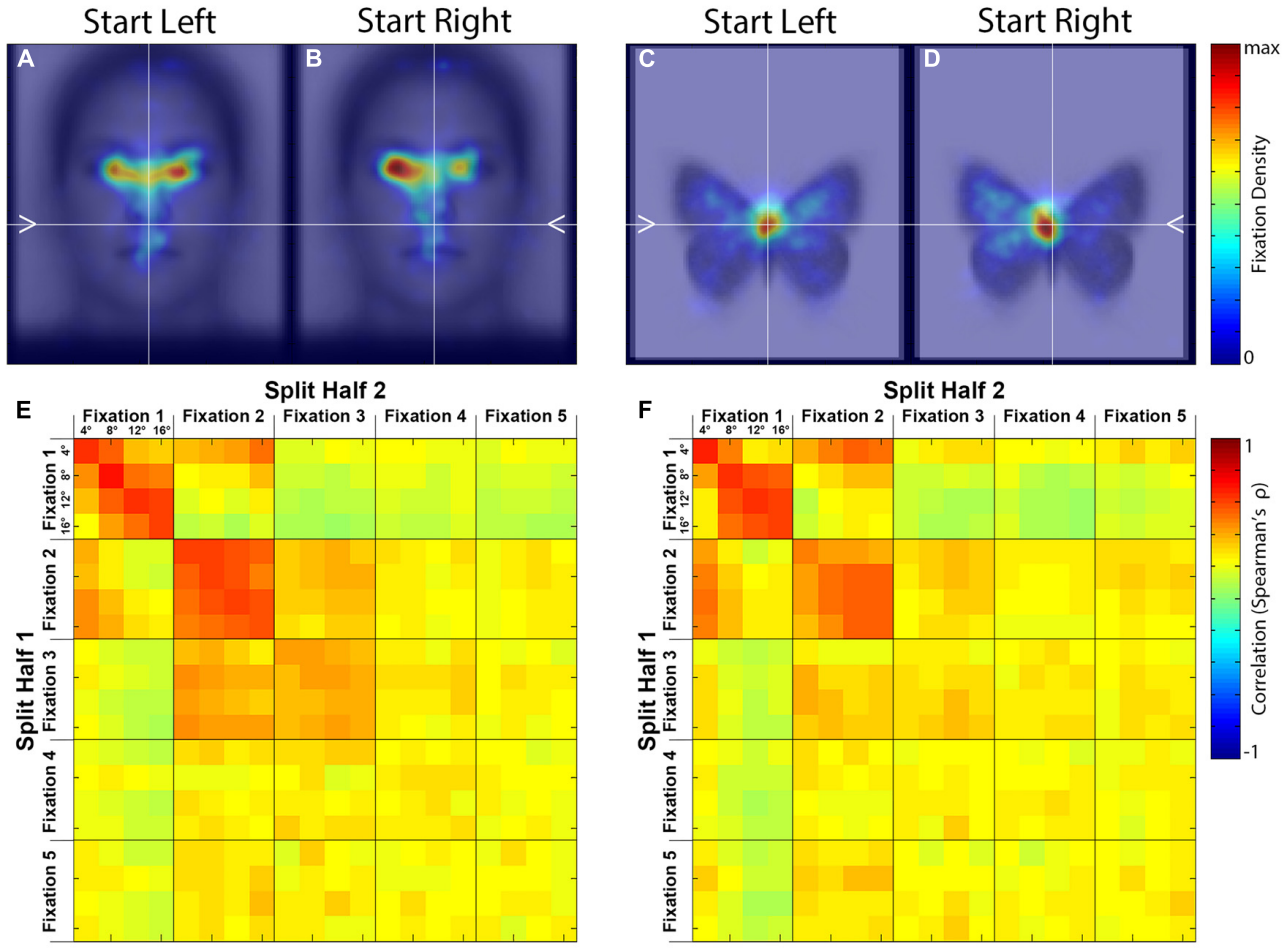
**Spatial density maps and similarity matrices. (A–D)** Spatial density maps by stimulus category and starting side. These densities reflect the second through fifth ordinal fixations pooled. **(E,F)** Split-half correlation matrices by ordinal fixation and starting distance for each stimulus category. The correlations are between spatial fixation densities.

To examine the overall pattern of fixations, we first produced spatial density plots broken down by Side and Category (**Figures 3A–D**), with the second through fifth Ordinal Fixations and all Distance conditions pooled. The first ordinal fixation was omitted because our prior study (Arizpe et al., 2012), as well as the current study, revealed that the first fixation is of a relatively short duration and thus is likely an orienting fixation, which is less meaningful in the current analysis. For faces, peak density of fixation occurred around the eye region, and for butterflies the peak density was close to the top of the main body. For both categories, an effect of Side is apparent, such that when the fixation dot was on the left of the upcoming stimulus there was a rightward tendency in overall fixation patterns, and conversely when the fixation dot was on the right there was the opposite overall tendency (see later sections for detailed quantitative analysis). This effect is consistent with our earlier study (Arizpe et al., 2012) suggesting that the influences of pre-stimulus start position are not specific to faces, but rather likely reflect a general visuomotor phenomenon. Further, because the location of the upcoming stimulus in each trial was much more unpredictable in the current study than in our prior study (Arizpe et al., 2012), both in terms of visual field relative to the starting fixation and also distance from the starting fixation, these results therefore indicate that the contralateral tendency in overall fixation patterns induced by start position seen in our prior study are, in principal, generalizable to situations of much greater uncertainty with respect to the where a target stimulus will appear.

#### Stimulus-Based Laterality in Spatial Density of Fixations

As an index of the lateral tendency in fixations to our stimuli, we calculated the proportion of spatial density to the left of midline, when left and right start positions and ordinal fixations two through five were again pooled. The result was greater than 50% on average for both faces (51.93% ± 4.12 SEM) and butterflies (59.08% ± 2.46 SEM) indicating a left-sided fixation tendency for both stimulus categories. A paired *t*-test between faces and butterflies on these left-sided proportions revealed that butterflies had a significantly greater left-sided fixation density proportion [paired *t*(13) = 2.36, *p* < 0.035, two-tailed, bias corrected *g*_Hedges_ = 0.53], indicating that butterflies may have had a more pronounced left-sided fixation tendency than faces, though the spatial density maps also indicate that peak density on average fell closer to midline for butterflies than for faces.

To test the correspondence between faces and butterflies in their lateral tendency in fixations, we performed a correlation between the two categories of stimuli on the proportions of spatial density of fixations to the left of midline (Side and Ordinal Fixations 2–5 pooled). This yielded a statistically significant positive correlation [*r*(13) = 0.72, *p* < 0.0027, two-tailed], indicating that individual differences in the lateral fixation tendency of one stimulus category directly related to that of the other stimulus category (see Supplementary Figure S8 for the scatterplot). Thus the overall group-level left-sided tendency in fixation, often reported for face stimuli, was not specific to faces, and the extent of the lateral bias in fixations for butterfly stimuli was related to that for faces.

#### Similarity of Fixation Patterns

To compare the fixation patterns across all conditions, we computed similarity matrices based on the spatial density plots (see Materials and Methods) containing comparisons between all levels of Distance (4°, 8°, 12°, 16° visual angle), Ordinal Fixation (1st, 2nd, 3rd, 4th, 5th), and Side (left, right) for each Stimulus Category (face, butterfly). Although there was an effect of side on the absolute fixation locations, there were no obvious differences using this relative similarity metric, therefore we pooled across this factor (**Figures 3E,F**).

First, the similarity matrices for faces and butterflies are remarkably similar, indicating that Distance and Ordinal Fixation influence fixation patterns in ways that are not specific to Stimulus Category. As will be apparent in subsequent analyses, the modulation by Distance is predominantly in the *x*-dimension. Second, correlations among conditions within the first two Ordinal Fixations are higher than those within the later fixations, and higher than across Ordinal Fixations, indicating a distinct fixation pattern for these two fixations. Third, for the first, and to some extent the second, Ordinal Fixation, correlations for the same Distance were higher than among different distances indicating an effect of Distance. There was not strong evidence for an effect of Distance on the later Ordinal Fixations.

### Discrimination Results

In order to test our hypothesis that Distance influences fixation patterns, and specifically to more quantitatively verify some of the suggested patterns in the similarity matrices just described, we conducted discrimination analyses (see Materials and Methods). Notably, Distance was fully discriminable (i.e., all four distances were significantly discriminable) only in the first ordinal fixation, for both faces [weakest discriminability among the four distances was *t*(13) = 2.93, *p* < 0.0059] and butterflies [weakest discriminability among the four distances was *t*(13) = 2.72, *p* < 0.0088]. In each of the later ordinal fixations, Distance was totally indiscriminable (i.e., not a single distance was significantly discriminable) with the exception the shortest distance in the second ordinal fixation for butterflies [*t*(13) = 4.33, *p* < 0.00042], and the longest distance in the fifth fixation for faces [*t*(13) = 2.26, *p* < 0.021]. This confirms that Distance influenced fixation patterns strongly only in the first ordinal fixation.

### Profile Analysis Results

**FIGURE 4:**
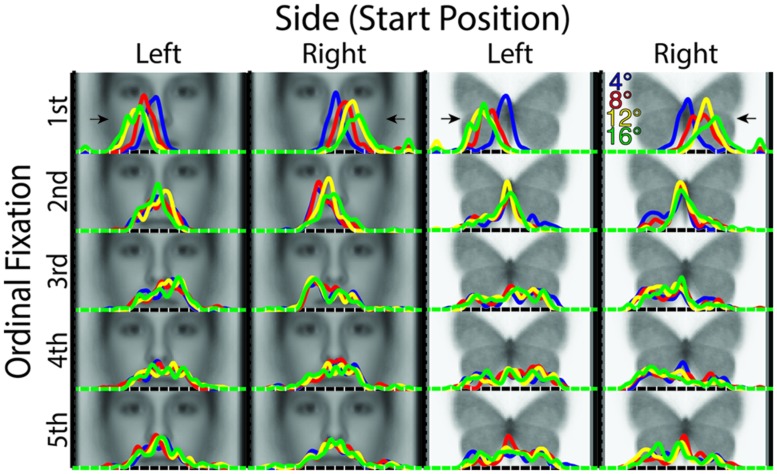
***X*-dimension profile density plots by stimulus category, starting distance, starting side, and ordinal fixation**. Of note is the increasing distance of fixations from midline of the stimulus in the first ordinal fixation with increasing starting distance. Also, there is a tendency for greater fixation density contralateral to the starting side overall following the first ordinal fixation. For reference, white tick marks along the *x*-axes indicate degrees of visual angle from stimulus midline.

Given our finding that Distance significantly modulated fixation patterns in the *x*-dimension in the first ordinal fixation, and the fact that what drives the similarities and discriminations in fixation patterns as seen in our prior analyses are not fully apparent without looking at the specific fixation patterns, we next closely examined profile density plots for the *x*- (**Figure 4**) and *y*-dimensions (Supplementary Figure S9).

The *x*-profile plots broken down by Ordinal Fixation, Distance, and Side (**Figure 4**) revealed a number of striking patterns, some of which, notably, were not detectable with our similarity matrix analyses. In brief, peak densities from the first ordinal fixation revealed an “undershoot” of the stimulus midline ipsilateral to the Side condition for all Distance conditions, but which was of a degree proportionate to the distance (i.e., longer Distance induced a greater “undershoot”). Third ordinal fixations tended to show an overall relative fixation density laterality that was contralateral to the Side condition for both faces and butterflies, but which appeared weakly, if at all, modulated by Distance, in accord with the discrimination analysis results already reported. The same seemed to hold true to some degree in the fourth ordinal fixation in butterflies, though otherwise, later fixations’ *x*-dimension densities tended to appear more variable in spread and less distinguishable among Side and Distance for both faces and butterflies.

The corresponding *y*-profile plots (Supplementary Figure S9) revealed, in accord with the patterns seen in the similarity matrices, that for faces, peak *y*-dimension density for the first ordinal fixation was slightly lower on the face than those of subsequent fixations, all of which had a peak in density just below the eyes. For butterflies, however, the peak *y*-dimension density did not seem to differ much across ordinal fixations, all of which had a peak just below the head or roughly near the convergence of the upper and lower wings. In general, for both faces and butterflies, fixation densities in the *y*-dimension did not seem to substantially differ between Side conditions, and the last three ordinal fixations seemed to become progressively more variable in spread in the *y*-dimension.

### Behavioral Measures

**FIGURE 5:**
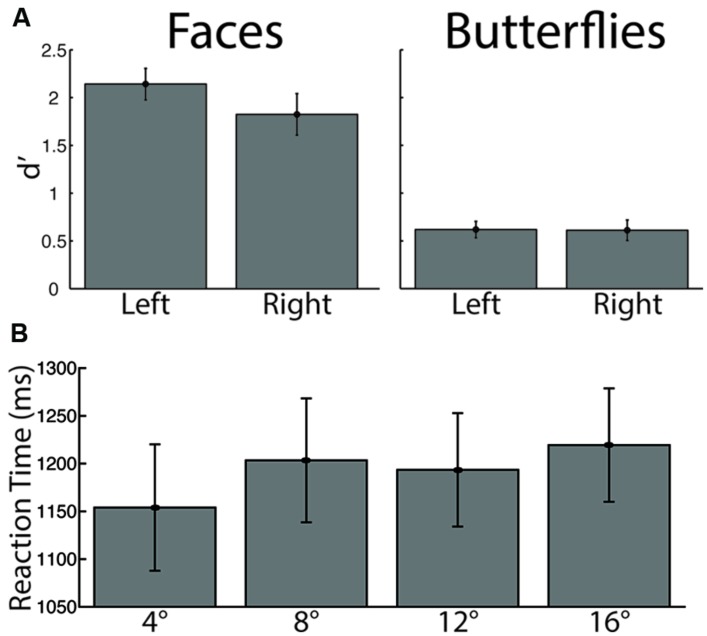
**Recognition performance by stimulus category and starting side. (A)** Discrimination performance, measured by *d*′. There was significantly lower discrimination for butterflies than for faces, and significantly greater face discrimination for the left than for the right starting side **(B)** Reaction time. The longest distance elicited significantly longer reaction times than the shortest distance.

In addition to the spatial and temporal dynamics of fixations just described, we additionally investigated whether our factors influenced recognition behavior. A three-way ANOVA on discrimination performance (*d*′) with Stimulus (faces, butterflies), Distance (4°, 8°, 12°, 16° of visual angle), and Side (left, right) as within-subject factors revealed a main effect of Stimulus [*F*(1,13) = 61.45, *p* < 0.0005, $\eta_{p}^{2}$ = 0.83], driven by overall higher discrimination performance for faces, but no main effects for either Distance or Side (both *p* > 0.19). No interactions reached significance (all *p* > 0.24). Given the quite low discrimination performance for butterflies overall and the hypothesis that Side would modulate face discrimination, we conducted additional planned *t*-tests on *d′* between Side conditions (left, right) for faces only. This yielded a significant difference in discrimination by Side [paired *t*(13) = 1.97, *p* < 0.036, one-tailed, bias corrected *g*_Hedges_ = 0.41], driven, as hypothesized, by higher discrimination performance for faces viewed starting on the left side. These results suggest that our participants found butterflies more difficult to discriminate than faces, and that faces were better discriminated when starting side was on the left (**Figure 5**).

A three-way ANOVA on criterion (c) again with Stimulus, Side, and Distance as within-subject factors was also conducted. No main effects or interactions yielded significant statistics (all *p* > 0.26), suggesting that these factors did not modulate response bias.

A three-way ANOVA on reaction time again with Stimulus, Side, and Distance as within-subject factors yielded a main effect of Distance [*F*(3,39) = 3.84, *p* < 0.018, $\eta_{p}^{2}$ = 0.23], driven by overall longer reaction time for the longest distance than the shortest distance [paired *t*(13) = 3.16, *p* < 0.005, bias corrected *g*_Hedges_ = 0.26] and possibly by a longer reaction time for the 8° distance than the 4° distance that approached statistical significance [paired *t*(13) = 2.06, *p* < 0.061, bias corrected *g*_Hedges_ = 0.19; all other comparisons, paired *t*(13) < 1.45, *p* > 0.17]. This is likely due, at least in part, to the fact that a longer saccade requires more time than a shorter one, which adds to the response time. No other main effects or interactions yielded significant statistics (all *p* > 0.096) from the three-way ANOVA.

## Discussion

### Effects of Distance, Side, and Stimulus Category

The principal aim of this study was to determine whether lateral starting Distance from a face impacts subsequent eye movement patterns. Since faces in real-life typically appear in peripheral vision and require an initial saccade to bring them close to the fovea, determining the impact of starting Distance from the face is particularly important for understanding eye movements to faces. We have previously demonstrated strong effects of Start Position (specifically up, down, left, right, and center of face) leading to an overall contralateral tendency in fixations and so in the present study we also manipulated the starting Side (i.e., left or right Start Position) to replicate and extend our prior findings (Arizpe et al., 2012). In particular we wanted to establish: (1) whether effects of starting side and distance are specific to faces, (2), whether the effects of starting side occur only when the location of the upcoming stimulus is predictable as in our original study and (3) whether any differences in fixation patterns associated with starting side and distance relate to behavioral performance. While we observed strong effects of Distance on the first ordinal fixation, with an increasing undershoot of the midline of the stimulus with increasing distance, there was no impact on later fixations. These effects of Distance, as well as the previously reported effects of Side, were not specific to faces. We also established that the subsequent contralateral tendency in fixation patterns does not depend on predictability of the location of the upcoming stimulus or on stimulus category (faces and butterflies). Lastly, we found evidence of a relationship between left starting Side (right-sided fixation pattern tendency) and increased recognition performance for faces.

### Effect of Distance in First Ordinal Fixation

We hypothesized that the location of the first fixation would systematically vary with distance. Given previous reports of a systematic saccadic range error for word and simple point stimuli (Kapoula, 1985; Kapoula and Robinson, 1986; McConkie et al., 1988; Radach and McConkie, 1998, but see Vitu, 1991), and taking the midline of the face or butterfly as the reference, we specifically hypothesized that we would find an overshoot of the midline for near stimuli and an undershoot for far stimuli. We did indeed find a systematic variation of the horizontal location of the first ordinal fixation as a function of lateral starting distance as evinced by the fact that all four distances were significantly discriminable from the fixation patterns in the first ordinal fixation; however, we did not find an overshoot for any distance, but rather an increasing undershoot of the midline of the stimulus with increasing distance as is apparent in the profile density plots (**Figure 4**). Individual distance conditions were largely not significantly discriminable in later fixations, so we did not find strong modulation of subsequent ordinal fixations as a function of distance. The influence of distance in the first ordinal fixation was, notably, not specific to faces, indicating visuo-motor factors that strongly influence eye-movement patterns independently of stimulus category and task factors.

### Effect of Starting Side Robust to Distance and Predictability

Overall fixation patterns tended contralateral to the start position (Side), replicating the effect of start position as reported in our prior study (Arizpe et al., 2012). Specifically, while the first fixation tended to fall ipsilateral to the start position, the later fixations tended contralateral. Importantly, this effect was not specific to faces. Rather, the asymmetry after the first fixation appeared even more pronounced for butterflies. Because the overall contralateral tendency in fixation patterns was present for all distances, even for the farthest distance, this contralateral effect of Side is unlikely to be due to presampling of stimulus information close to (ipsilateral to) the start position, since little or no presampling of the stimulus could occur at the farther starting distances. Of note, the location of the upcoming stimulus, with respect to the start position, was much more unpredictable in the current study than in our prior study, both in terms of distance and visual field. Thus, the current study clarifies that the contralateral effect of start position was not dependent on the predictability of the location of the upcoming stimulus. The correspondence in the effects of Side on eye-movement patterns between faces and butterflies, further, suggests that visuomotor influences on eye-movements are general across stimulus categories.

### Eye-Movement Relationship to Behavior

We also investigated whether the differences in laterality of overall fixation patterns we observed were related to recognition performance. Distance did not significantly modulate the degree of contralateral tendency induced by starting Side, and so Distance conditions could be pooled to compare the effect of Side on recognition performance. It has previously been suggested that faces tend to be recognized better when presented in the left visual field (Hellige et al., 1984; Luh et al., 1991, 1994; Dutta and Mandal, 2002, though see Sergent and Bindra, 1981; Sergent, 1982; Hellige et al., 1984; Rhodes, 1985), which likely reflects some right hemispheric specialization in the brain for face identity representation (Yovel et al., 2008), as suggested in split-brain (Levy et al., 1972), neuropsychological (Warrington and James, 1967; Benton and Van Allen, 1968; Sergent et al., 1992b; De Renzi et al., 1994), PET/fMRI (Sergent et al., 1992a,b; Kanwisher et al., 1997; Kanwisher and Yovel, 2006; Chan et al., 2010), and electrophysiological (Bentin et al., 1996; Campanella et al., 2000; Rossion et al., 2003; Yovel et al., 2003) studies on humans. Intriguingly though, a bias for fixating on the left side of the face has also been reported when free viewing was allowed (Gallois et al., 1989; Mertens et al., 1993; Phillips and David, 1997; Butler et al., 2005; Everdell et al., 2007; Guo et al., 2009, 2010, 2012; Saether et al., 2009; van Belle et al., 2010). Given that start position strongly modulates the laterality of fixation patterns, it could be hypothesized that it therefore also modulates recognition performance. In our prior study (Arizpe et al., 2012), we found a trend for higher discrimination performance with a left start position; however, several aspects of the design may have prevented strong detection of such effects. Specifically, left and right start positions were swapped between study and test phases for half of the trials, there were limited trials per subject for each start position for upright faces, and participants were allowed up to 10 s to study each face. In the current study, the first two limitations did not exist, and also participants had a more restricted time (up to 5 s) during which they could study the stimuli. With these changes in design, we did find significant (*p* < 0.036, one-tailed) higher discrimination performance for when faces were viewed with a left sided-start position, which corresponds to a right-sided tendency in fixation patterns. When fixating on the right side of a face, most of the face is in the left visual field, thus this result is consistent with a right-hemisphere cortical advantage for face perception and representation. Though the first ordinal fixation tended to fall ipsilateral to the start position, it had a shorter duration relative to later fixations, regardless of distance, and there is evidence that stimulus information is not deeply processed in just the first fixation (Renninger et al., 2007; Hsiao and Cottrell, 2008). Thus we suspect that there was shallow sampling of the side of the stimulus ipsilateral to the start position in the first ordinal fixation, and that this difference in performance is driven by the fixations contralateral to the start position. We did not find any evidence of difference for butterflies, though discrimination performance was overall quite low for butterflies. These data regarding the relationship between eye-movements and behavior are suggestive and preliminary, and so further research is warranted to better characterize how eye-movement patterns relate to visual recognition performance and information use (Luh et al., 1991, 1994; Burt and Perrett, 1997; Butler et al., 2005; Malcolm et al., 2008; Samson et al., 2014), whether such effects are specific to faces (Leonards and Scott-Samuel, 2005), and how such relationships may be subject to individual differences (Levine et al., 1988; Miellet et al., 2011; Peterson and Eckstein, 2013; Mehoudar et al., 2014).

### Differences in Processing between Butterflies and Faces

As already stated, we observed a close correspondence between faces and butterflies in the similarities in influences of Distance and Side on spatial fixation patterns across ordinal fixations. We did, however, also observe lower discrimination performance and longer short distance latencies to first saccade for butterflies than faces, suggesting the stimulus information of butterflies required more effort to process and was more difficult to recognize. Additionally, while our similarity measures indicate that the influences of Distance and Side are highly correspondent between faces and butterflies, nonetheless, the fixation patterns for the two stimuli categories are quite distinct, just by virtue of the stimulus categories being different. Though the patterns of information contained within our butterfly stimuli are arguably much more distinct than faces from a computer vision perspective (each butterfly image was of a distinct species of Lepidoptera, not separate individuals within a species), our participants nonetheless seem to have found butterflies more difficult to visually process and discriminate. This likely reflects specialized visual processing mechanisms and expertise for facial recognition.

### Methodological Implications for Eye-Tracking Studies

Our findings can inform the design and analytic considerations of eye-tracking studies of visual perception. The strong influence of Distance in the first ordinal fixation, but not for subsequent fixations, suggests that for eye-movement studies in which the experimental factors of interest are stimulus- or task-related, lateral starting distance can be safely modulated without the danger of strongly confounding visuomotor influences on eye-movements beyond the first fixation. This result, together with the significantly shorter duration of the first ordinal fixation and prior evidence that the visual information is not deeply processed in the first fixation (Renninger et al., 2007; Hsiao and Cottrell, 2008), indicates that when analyzing eye-movements, it may be a good general practice to exclude the first ordinal fixation.

The overall tendency for fixations to fall on stimulus regions contralateral to the starting side (after the first ordinal fixation), regardless of the stimulus category, indicates that there are general visuomotor factors that must be controlled for in the design of studies of eye-movements and considered in the analysis and interpretation of eye-movement data. This contralateral tendency in fixation patterns is strongly present whether the location of the upcoming stimulus is predictable (Arizpe et al., 2012) or is unpredictable, as in the current study; therefore, such visuomotor influences cannot be eliminated through modulation of the predictability of the location of stimuli, and must simply be taken into account when interpreting eye-movement data.

The kind of correlation matrix and discrimination analyses utilized in the present study may, when possible and relevant, be an advantageous set of analysis methods for detecting the independent effects of stimulus- and task-related factors even though potentially confounding visuomotor factors are also present in a study. This is because correlation matrices containing the factors of interest can simply be averaged across the separate matrices for each start position condition. These factors out any potential nuisance visuomotor influences from subsequent discrimination analyses without the artificial regressing of fixation patterns to the mean between start position conditions that would otherwise occur with the common practice of averaging spatial density maps. More broadly though, the additional potential for hypothesis generation and for data-driven approaches from such analysis methods could be of great utility in eye-tracking studies of visual perception.

Many studies investigating eye movements to faces, only test faces themselves, making it unclear whether any effects, where relevant, are specific to faces or not. Our finding of influences on eye-movements not only for faces, but also for butterflies, highlights the importance of including control stimuli in eye-movement experiments so that inferences regarding stimulus specificity or generality can be made.

### Mechanistic Implications for Visual Perception and Further Questions

We report that the overall tendency to fixate the left side of the stimulus was not specific to faces, but was also present, and apparently to a greater extent, for our line-symmetric stimulus category, butterflies. Indeed, a significant positive correlation [*r*(13) = 0.72, *p* < 0.0027, two-tailed] between faces and butterflies on the proportions of spatial density of fixations to the left of midline indicate that our participants’ individual differences in the lateral fixation tendency of one stimulus category directly related to that of the other stimulus category. Thus the extent of the lateral bias in fixations for butterfly stimuli was related to that for faces. Though the left-sided fixation tendency is often noted in face perception, and thought to reflect face specific mechanisms, there is the possibility that this tendency may be general to all other stimulus categories. This notion is consistent with some prior research (Levine et al., 1988; Luh et al., 1991, 1994), and is worth further investigation to elucidate the general mechanisms of visual recognition and the specific mechanisms of face recognition. Visual representation of faces tends to be cortically right-hemisphere lateralized (Warrington and James, 1967; Benton and Van Allen, 1968; Levy et al., 1972; Sergent et al., 1992a,b; De Renzi et al., 1994; Bentin et al., 1996; Kanwisher et al., 1997; Campanella et al., 2000; Rossion et al., 2003; Yovel et al., 2003, 2008; Kanwisher and Yovel, 2006; Chan et al., 2010), but there is evidence that representations for other stimulus categories may tend to be right hemisphere lateralized as well (Warrington and Taylor, 1973; Warrington and James, 1986; Konen et al., 2011) and also that perceptual asymmetries may relate to asymmetric hemispheric arousal (Levy et al., 1983), and so the left-sided tendency in fixation may be related to this characteristic functional neuroanatomy.

Relatedly, our recognition performance results indicate a paradox, which reveals that the relationship between lateralized fixation tendency, hemispheric lateralization, and behavioral performance requires much deeper investigation. We found a significantly higher discrimination performance for when faces were viewed with a left sided-start position, which corresponds to an overall right-sided tendency in fixation patterns (after the first ordinal fixation). When fixating on the right side of a face, most of the face is in the left visual field. Thus, improved recognition performance under this condition is consistent with a right-hemisphere cortical advantage for face perception and representation. However, if recognition performance is indeed more optimal for right-sided fixation on the faces, then it is puzzling why there is the natural tendency to fixate the left side of the face, as has been ubiquitously reported in prior studies (Gallois et al., 1989; Mertens et al., 1993; Phillips and David, 1997; Butler et al., 2005; Everdell et al., 2007; Guo et al., 2009, 2010, 2012; Saether et al., 2009; van Belle et al., 2010), and how this relates to the tendency to use the information on the left side of the face during perceptual judgment (Gilbert and Bakan, 1973; Luh et al., 1991, 1994; Burt and Perrett, 1997; Butler et al., 2005). Though recognition performance was overall too low for butterflies to determine if a similar asymmetry in performance existed for butterflies, there is the possibility that such a paradox applies for visual stimulus categories besides faces too. It is unclear what factors may induce eye-movement patterns which are less than optimal for the recognition to be employed during recognition tasks.

Though there was an influence of Side present throughout the first five fixations, the lack of a strong independent influence of Distance past the first fixation is intriguing. It suggests that the first fixation may reflect a simple initial localizing saccade to the stimulus, required before a more stereotyped information sampling program of fixations can be employed. This reveals the limits of the non-stimulus non-task visuomotor influences on eye-movements, and thereby suggests that definite loci on stimuli serve as functional targets for visual information extraction, the visuomotor influences (Distance and Start Position) notwithstanding.

## Conclusion

We investigated the effects of lateral starting distance and side from a face on subsequent eye movement patterns, and whether such effects may generalize to other line symmetrical stimuli (butterflies). We found an increasing undershoot of the midline of the stimulus with increasing distance for the first ordinal fixation, which was not specific to faces. A tendency for later fixations to fall contralateral to the left- or right-lateralized start position was observed as in our previous study (Arizpe et al., 2012), and this did not depend on the predictability of the location upcoming stimulus. Lastly, we found preliminary evidence for a relationship between left starting position (right-sided fixation pattern tendency) and increased recognition performance for faces that deserves further investigation.

## Author Contributions

Conception and design: JA, VW, CB. Acquisition and analysis of data: JA, CB. Interpretation of analyses and composition of the paper: JA, VW, CB.

## Conflict of Interest Statement

The authors declare that the research was conducted in the absence of any commercial or financial relationships that could be construed as a potential conflict of interest.

## Footnotes

1 http://vitallongevity.utdallas.edu/stimuli/facedb/categories/neutral-faces.html

2 http://www.butterfliesofamerica.com
