# Supplementary material for: Characteristic visuomotor influences on eye-movement patterns to faces and other high level stimuli
Source: Front Psychol. 2015 Jul 29;6:1027. doi: 10.3389/fpsyg.2015.01027 (PMC4518262; doi:10.3389/fpsyg.2015.01027)
Supplement: Supplementary file 1 [file Data_Sheet_1.DOCX]

*Supplemental Material*

Characteristic visuomotor influences on eye-movement patterns to faces and other high level stimuli

Joseph Arizpe*^1,2,3,4^, Vincent Walsh^1^, Chris I. Baker^2^

^1^Applied Cognitive Neuroscience Group, Institute of Cognitive Neuroscience, University College London, London, United Kingdom

^2^Section on Learning and Plasticity, National Institute of Mental Health, National Institutes of Health, Bethesda, MD, USA

^3^Neurology Department, University of Tennessee Health Science Center, Memphis, TN, USA

^4^Pediatrics Department, Le Bonheur Children's Hospital, Memphis, TN, USA

***Correspondence:** Joseph Arizpe, Applied Cognitive Neuroscience Group, Institute of Cognitive Neuroscience, University College London, 17 Queen Square, London, WC1N 3AR, United Kingdom

Joseph.Arizpe.10@ucl.ac.uk

**1. Supplementary Figure 1**


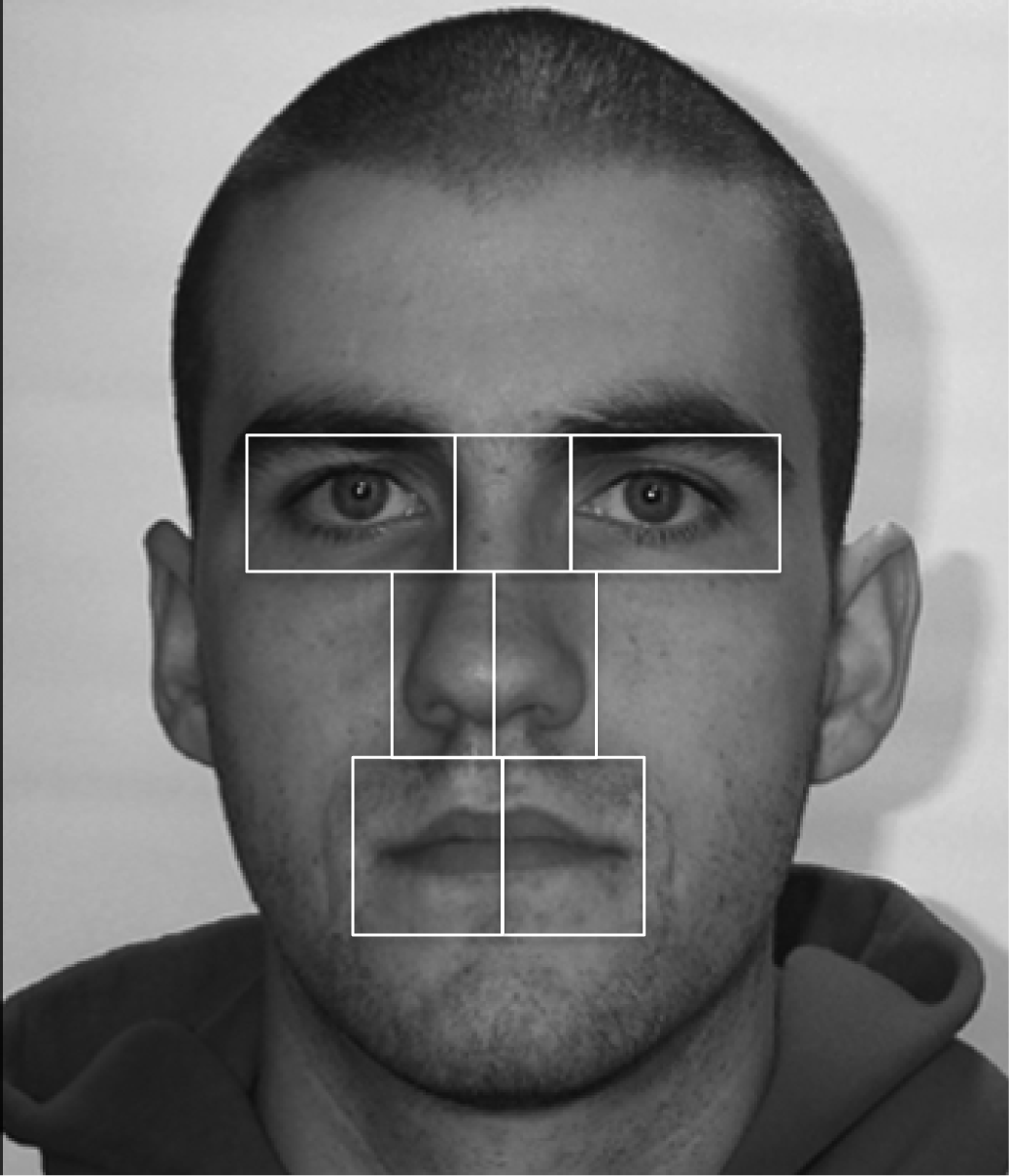


**Supplemental Figure 1. Example Areas of Interest (AOIs).** AOIs are shown in white. These AOIs were used only for determining the initial start position during stimulus presentation and for stimulus alignment during analysis. AOIs were not visible to the participants.

**2. Supplementary Figure 2**


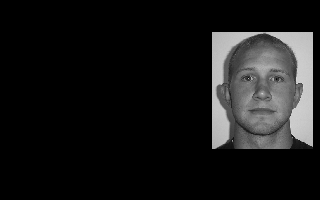


**Supplemental Figure 2. Full Screen Example of Stimulus: Start 16° Left (of Face Midline).** Figures showing example stimuli among the main figures showed only portions of the full screen stimuli. Here we show a full screen example of a stimulus presentation, as a participant would have seen it, on a trial displaying a male face for the start 16° Left (of face midline) condition. Immediately prior to stimulus presentation, participants were required to fixate at a dot at the center of the screen.

**3. Supplementary Figure 3**


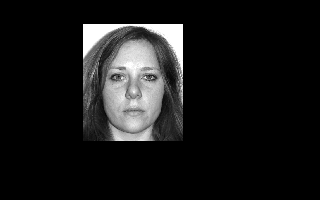


**Supplemental Figure 3. Full Screen Example of Stimulus: Start 4° Right (of Face Midline).** Figures showing example stimuli among the main figures showed only portions of the full screen stimuli. Here we show a full screen example of a stimulus presentation, as a participant would have seen it, on a trial displaying a female face for the start 4° Right (of face midline) condition. Immediately prior to stimulus presentation, participants were required to fixate at a dot at the center of the screen.

**4. Supplementary Figure 4**


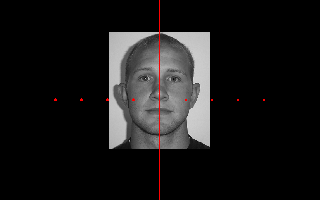


**Supplemental Figure 4. Schematic of All Possible Start Positions (Relative to an Example Face).** During the experiment, the locations of the stimuli varied with respect to a fixed central pre-stimulus start position. Here we show a full screen sized schematic, in a stimulus-centered space, of possible start positions relative to an example stimulus. Red dots indicate all of the possible (relative) start positions, and a red line indicates the midline of the stimulus.

**5. Supplementary Figure 5**


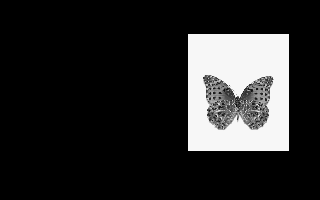


**Supplemental Figure 5. Full Screen Example of Stimulus: Start 12° Left (of Butterfly Midline).** Figures showing example stimuli among the main figures showed only portions of the full screen stimuli. Here we show a full screen example of a stimulus presentation, as a participant would have seen it, on a trial displaying a butterfly for the start 12° Left (of butterfly midline) condition. Immediately prior to stimulus presentation, participants were required to fixate at a dot at the center of the screen.

**6. Supplementary Figure 6**


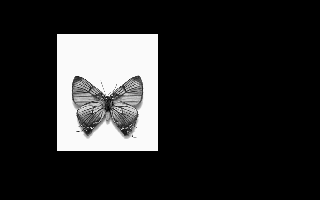


**Supplemental Figure 6. Full Screen Example of Stimulus: Start 8° Right (of Butterfly Midline).** Figures showing example stimuli among the main figures showed only portions of the full screen stimuli. Here we show a full screen example of a stimulus presentation, as a participant would have seen it, on a trial displaying a butterfly for the start 8° Right (of butterfly midline) condition. Immediately prior to stimulus presentation, participants were required to fixate at a dot at the center of the screen.

**7. Supplementary Figure 7**


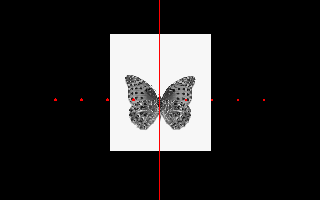


**Supplemental Figure 7. Schematic of All Possible Start Positions (Relative to an Example Butterfly).** During the experiment, the locations of the stimuli varied with respect to a fixed central pre-stimulus start position. Here we show a full screen sized schematic, in a stimulus-centered space, of possible start positions relative to an example stimulus. Red dots indicate all of the possible (relative) start positions, and a red line indicates the midline of the stimulus.

**8. Supplementary Figure 8**


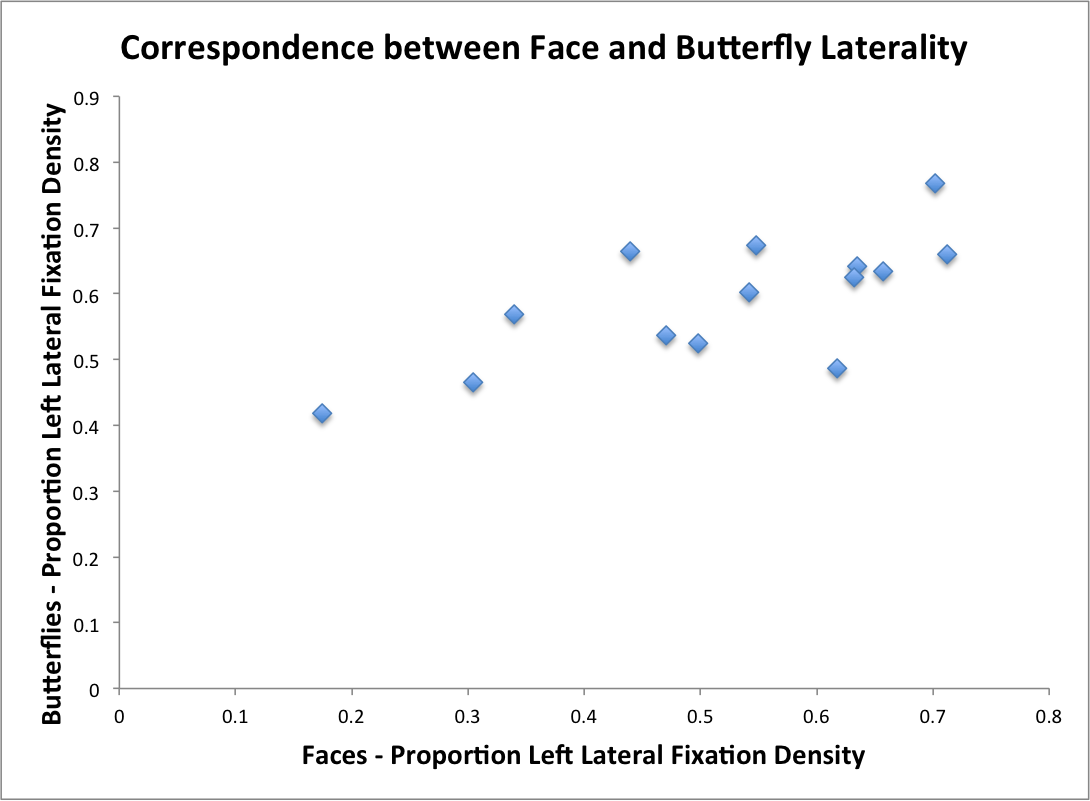


**Supplemental Figure 8. Scatterplot of Face versus Butterfly Left Lateral Fixation Density.** Individual differences in proportions of left lateral fixation density correlate between face and butterfly stimuli (r(13) = 0.72, p < 0.0027, two-tailed).

**9. Supplementary Figure 9**


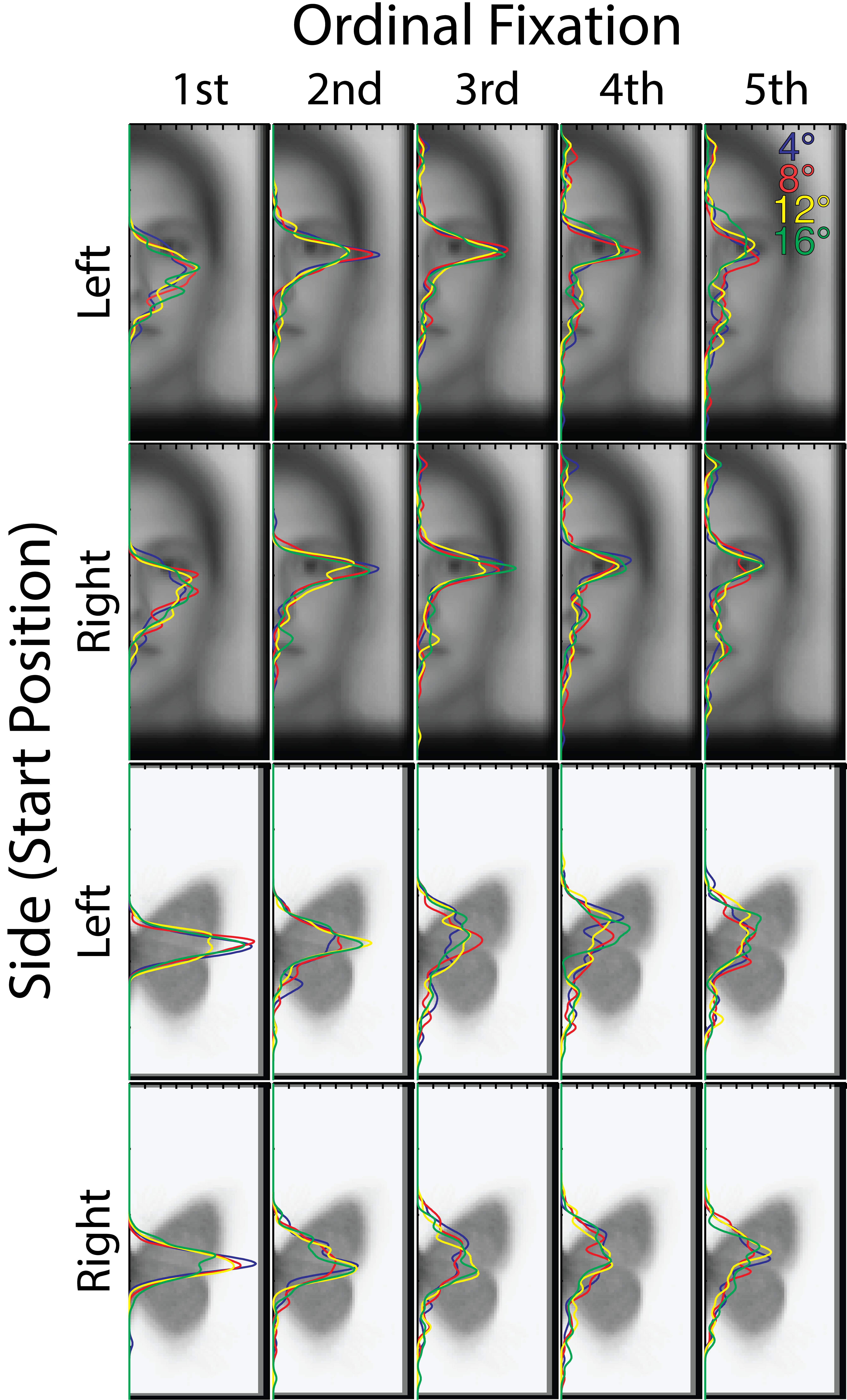


**Supplemental Figure 9. Y-dimension profile density plots by stimulus category, starting distance, starting side, and ordinal fixation.** Starting side and distance did not appear to modulate y-dimension profile density, though ordinal fixation did.
